# Rosemary Extract Inhibits Proliferation, Survival, Akt, and mTOR Signaling in Triple-Negative Breast Cancer Cells

**DOI:** 10.3390/ijms21030810

**Published:** 2020-01-27

**Authors:** Alina Jaglanian, Evangelia Tsiani

**Affiliations:** 1 Department of Health Sciences, Brock University, St. Catharines, ON L2S 3A1, Canada; aj11fo@brocku.ca; 2 Centre for Bone and Muscle Health, Brock University, St. Catharines, ON, L2S 3A1, Canada

**Keywords:** rosemary extract, breast cancer, proliferation, survival, apoptosis, signaling

## Abstract

Breast cancer is the most commonly diagnosed cancer in women. Triple-negative (TN) breast cancer lacks expression of estrogen receptor (ER), progesterone receptor (PR) as well as the expression and/or gene amplification of human epidermal growth factor receptor 2 (HER2). TN breast cancer is aggressive and does not respond to hormone therapy, therefore new treatments are urgently needed. Plant-derived chemicals have contributed to the establishment of chemotherapy agents. In previous studies, rosemary extract (RE) has been found to reduce cell proliferation and increase apoptosis in some cancer cell lines. However, there are very few studies examining the effects of RE in TN breast cancer. In the present study, we examined the effects of RE on TN MDA-MB-231 breast cancer cell proliferation, survival/apoptosis, Akt, and mTOR signaling. RE inhibited MDA-MB-231 cell proliferation and survival in a dose-dependent manner. Furthermore, RE inhibited the phosphorylation/activation of Akt and mTOR and enhanced the cleavage of PARP, a marker of apoptosis. Our findings indicate that RE has potent anticancer properties against TN breast cancer and modulates key signaling molecules involved in cell proliferation and survival.

## 1. Introduction

Breast cancer is the most frequently diagnosed cancer in women, accounting for over 1.3 million diagnosed cases annually worldwide [1]. It is estimated that roughly 20% of all diagnosed breast cancer cases will display the triple-negative (TN) phenotype [2,3], characterized by the lack of expression of estrogen (ER) and progesterone (PR) receptors and lack of expression and/or gene amplification of human epidermal growth factor receptor 2 (EGFR2/HER2) [2,3]. TN breast cancer has the worst prognosis of all breast cancer subtypes, tends to relapse earlier and more frequently, shows an early pattern of metastasis, and generally has a short progression-free survival time [2]. TN breast cancer is aggressive, does not respond to hormone therapy, and has limited treatment options; therefore, innovative treatments are urgently needed [4].

Cancer cells are characterized by their ability to proliferate uncontrollably and evade apoptosis [5,6]. These characteristics are often acquired as a result of mutations in key proteins involved in the signaling pathways responsible for regulating cell function and maintaining homeostasis [7,8,9,10]. Molecular signaling pathways of growth factor receptors [9], such as epidermal growth factor receptor (EGFR/HER1) initiate signal transduction pathways [11] including the phosphatidylinositol 3-kinase (PI3K)/Akt [10,12,13,14,15,16] and the Ras/mitogen-activated protein kinase (MAPK) pathways [17,18], which lead to increased cell proliferation and survival. PI3K is a lipid kinase and, when activated, phosphorylates membrane phospholipids, resulting in the generation of 3-phosphoinositides, mainly phosphatidylinositol-3,4,5-triphosphate (PIP3). PIP3 contributes to the activation of the serine/threonine protein kinase Akt, a key promoter of cell proliferation and survival [10,13,14]. Activated Akt leads to activation of mechanistic target of rapamycin (mTOR) and p70S6K, which increase protein synthesis and cell proliferation [10,13,14]. In addition, activated Akt phosphorylates and inhibits the proapoptotic Bcl-2 family members Bad and Bax, resulting in an inhibition of apoptosis and increased cell survival [19,20]. In many types of cancer the Akt pathway is upregulated as a result of genetic alterations that lead to signaling activation even in the absence of growth factors [11]. It is well established that overactivation of the PI3K/Akt pathway contributes to tumorigenesis [11,15,21]. Mutated Akt can function as an oncogene and increase tumor aggressiveness, while the proteins involved in inhibiting the Akt pathway such as phosphatase and tensin homolog (PTEN) function as tumor suppressors [21]. Studies analyzing human tumors have shown that at least 33% of TN breast cancers have dysregulated PI3K/Akt pathways and 11.3%–35% have a mutation/loss-of-function of the PTEN gene [22,23]. Additionally, Akt overexpression is associated with increased resistance to chemotherapeutic agents such as cisplatin, methotrexate, or paclitaxel [16,21].

The serine/threonine kinase mTOR is a downstream target of Akt, leading to protein synthesis, cell proliferation and survival [24,25,26]. mTOR activation leads to enhanced translation of mRNA encoding proteins essential for cell growth and cell cycle progression [27]. Enhanced activation of mTOR can be caused by either the amplification of the *PI3K* gene, the mutation of Akt, or the loss of function of PTEN [28,29]. mTOR signaling is overactivated in many types of cancer [25] including breast, ovarian, renal, colon, and head and neck cancers [24]. Overactivated mTOR signaling in breast cancer is linked to poor prognosis and decreased patient survival [27,30,31,32]. In triple-negative breast cancer specifically, an increased expression of phosphorylated mTOR has been reported [33]. Due to the importance of these signaling proteins, several small molecules that target/inhibit Akt [34,35,36], mTOR [37,38], or both are currently in clinical development.

Alongside increased cell proliferation and survival, evasion of apoptosis is another key hallmark of cancer cells. The poly ADP-ribose polymerase (PARP) family of proteins play a key role in cell apoptosis. PARP-1 is an enzyme responsible for roughly 90% of ADP-ribosyl transferase activity [39,40]. PARP enzymatic function is activated in response to DNA damage. When the damage is repairable, PARP-1 regulates cell survival, however, when DNA damage cannot be repaired, PARP is cleaved into fragments that inactivate the enzyme by destroying its ability to respond to DNA strand breaks, thus inducing cell death [39,40]. PARP activation helps cells maintain their viability, while cleaved PARP is a known indicator of cell apoptosis, as it promotes cellular disassembly [39,40].

Cancer cells also display increased metastatic and invasive capabilities as a result of genetic changes during oncogenesis [11,17,41]. Typically, breast cancer will exhibit an expression of estrogen and progesterone receptors and an amplification of HER2 [3]. These markers allow for breast cancer tumors to be classified as hormone receptor positive (luminal A or B), HER2 overexpressing, or TN breast cancers, which do not express ER and PR and do not have HER2 amplification [3]. Tumors that express hormone receptors (estrogen and progesterone) are generally treated with agents that interfere with hormone production or inhibit ER signaling [3,42]. These tumors tend to have a more favorable outcome when compared to tumors with HER2 amplification or TN breast cancers [3]. Tumors that express HER2 amplification are treated most commonly with tyrosine kinase inhibitors [28,29]. Despite the absence of hormone and HER2 receptors in TN breast cancers, the signaling pathways that regulate cell survival and proliferation remain in an over-activated state. The use of hormone therapy or HER2 therapy in TN breast cancer is ineffective and thus there are no targeted therapies used for this sub-class of breast cancer specifically [2]. Studies have shown that while triple-negative breast cancer may respond well to primary chemotherapeutic agents such as taxane- or anthracycline-based therapies, there is a high risk of relapse [2].

Many agents that are used for cancer treatment have been derived from plants [43,44]. For example, the established chemotherapeutics paclitaxel and docetaxel were originally isolated from the bark of the Pacific yew (*Taxus brevifolia*) [45]. Recently, plant-derived extracts with high polyphenolic content such as green tea [46], rosemary extract [47], and individual polyphenols, such as quercetin [48], resveratrol [49,50,51], oleuropein [52], and others, have also shown anticancer effects. Rosemary extract (RE) from the plant *Rosmarinus officinalis* contains many chemicals including the polyphenols carnosic acid (CA), rosmarinic acid (RA), and carnosol (COH) found in high concentrations [53,54,55]. RE and RE polyphenols have been reported to have antioxidant and antimicrobial properties [56]. We have previously prepared a methanol-based extract of rosemary leaves in our lab and when tested in lung cancer cells, we found a significant inhibition of proliferation and survival as well as an inhibition of Akt, mTOR, and p70S6K [57]. A review of the literature revealed extensive evidence of the anticancer effects of RE and RE polyphenols [47]. The environmental conditions such as soil quality, sun exposure, and water availability may influence the levels of different chemicals/polyphenols in a plant, including rosemary. Furthermore, the extraction method may also influence the levels of chemicals in an extract. Despite these considerations, the scientific evidence points to consistent anticancer properties of RE [47]. A limited number of studies have found that, in various breast cancer cells, RE can decrease cell viability, inhibit cell proliferation, induce apoptosis, and enhance the effects of chemotherapeutic drugs [58,59,60,61]. However, the effects of RE in triple-negative breast cancer have not been well investigated.

In the present study we investigated the effects of RE on MDA-MB-231 TN breast cancer cell proliferation and survival/apoptosis. We also examined the effects of RE on Akt and mTOR signaling.

## 2. Results

### 2.1. Effects of Rosemary Extract on MDA-MB-231 Breast Cancer Cell Proliferation

**Figure 1:**
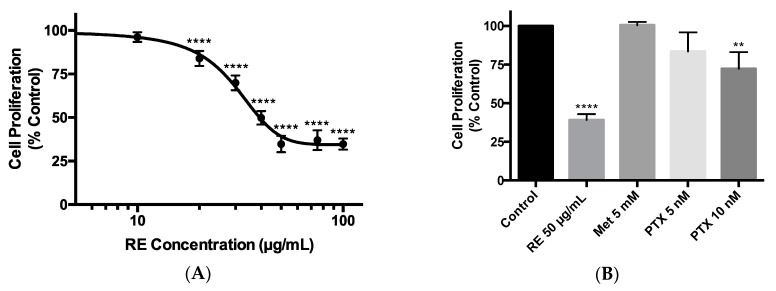
Effect of rosemary extract on MDA-MB-231 breast cancer cell proliferation. MDA-MB-231 cells were treated with 10, 20, 30, 40, 50, 75, or 100 µg/mL of rosemary extract (RE) (**A**,**B**), metformin (Met) (**B**), or paclitaxel (PTX) (**B**) for 72 h, followed by fixing and staining with 0.5% crystal violet. The stain was solubilized, and absorbance was read at 570 nm. Data are expressed as percent of control, untreated cells. Data are the mean ± SEM of six independent experiments. ** *p* < 0.01, **** *p* < 0.0001.

The triple-negative MDA-MB-231 breast cancer cells were exposed to 10, 20, 30, 40, 50, 75, or 100 µg/mL RE for 72 h and cell proliferation was assessed using the crystal violet assay. We dissolved the RE powder in dimethyl sulfoxide (DMSO) to create a 100 mg/mL stock solution. From that we created a working stock of RE (400 µg/mL) using cell culture media, which was used to treat the cells. Treatment with RE resulted in a dose-dependent inhibition of cell proliferation. A significant inhibition (83.95% ± 1.96% of control, *p* < 0.0001) was seen with 20 µg/mL RE while maximum inhibition (34.79% ± 2.32% of control, *p* < 0.0001) was seen with 50 µg/mL RE (Figure 1A,B). Higher RE concentrations (75 and 100 µg/mL) did not result in any greater inhibition of cell proliferation (Figure 1A). The final concentration of DMSO in the RE treated cells was less than 0.1%. Exposure of the cells to DMSO to match the concentration of DMSO seen by cells exposed to RE (vehicle control) did not have any effect on cell proliferation (data not shown). The data in Figure 1A were plotted using a log scale of RE concentrations and the calculated concentration of RE for half maximal inhibition (IC_50_) of cell proliferation was 28.86 µg/mL. Previous studies by our group [62,63] and others [64,65] have shown anticancer effects of metformin, a drug used to treat hyperglycemia /type 2 diabetes mellitus [66], and we used metformin in the present study to compare its effects with the effects of RE. In addition, paclitaxel, derived from *Taxus brevifolia*, is an established medication used clinically in the treatment of triple-negative breast cancer [67,68], and we used it in the present study to compare the effects of RE to the effects of paclitaxel. Treatment of the cells with 5 mM metformin did not result in any significant inhibition of cell proliferation (100.5% ± 2.10% of control) (Figure 1B), while treatment with 10 nM of paclitaxel showed significant inhibition of cell proliferation (72.31% ± 10.77% of control, *p* < 0.01) (Figure 1B). The inhibition of cell proliferation seen with 50 µg/mL of rosemary extract (34.79% ± 2.32% of control, *p* < 0.0001) was greater than that seen with 10 nM of paclitaxel (72.31% ± 10.77% of control, *p* < 0.01).

### 2.2. Effects of Rosemary Extract on MDA-MB-231 Breast Cancer Cell Survival

**Figure 2:**
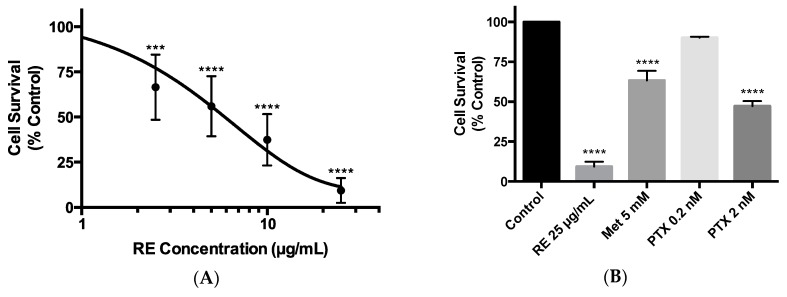
Effect of rosemary extract on MDA-MB-231 breast cancer cell survival. MDA-MB-231 cells were seeded (1000 cells/well) in six-well plates and exposed to 2.5, 5, 10, or 25 µg/mL of rosemary extract (RE) (**A**,**B**), metformin (Met) (**B**), or paclitaxel (PTX) (**B**) for 7 days followed by fixing and staining with 0.05% methylene blue. Colonies of more than 50 cells were counted. Data are expressed as percent of control, untreated cells. Data are the mean ± SEM of six independent experiments. *** *p* < 0.001, **** *p* < 0.0001.

The ability of cancer cells to survive and form colonies was assessed in a clonogenic survival assay. Exposure of MDA-MB-231 cells to 2.5, 5, 10, or 25 µg/mL of RE resulted in a concentration-dependent inhibition of survival. The lowest concentration of RE that resulted in a significant inhibition of cell survival was 2.5 µg/mL (66.47% ± 7.39% of control, *p* < 0.001). The greatest inhibition (9.33% ± 3.09% of control, *p* < 0.0001) of cell survival was seen at 25 µg/mL RE (Figure 2A,B). The data in Figure 2A were plotted using a log scale of RE concentrations and the calculated RE concentration for the half maximal inhibition (IC_50_) of cell survival was 4.82 µg/mL. Exposure of the cells to 5 mM of metformin was found to significantly inhibit MDA-MB-231 cell survival (63.36% ± 6.01% of control, *p* < 0.0001) (Figure 2B). In addition, treatment with 2 nM of paclitaxel resulted in a significant inhibition of cell survival (47.28% ± 3.13% of control *p* < 0.0001). It should be noted that the inhibition of MDA-MB-231 cell survival seen with 25 µg/mL of RE (9.33% ± 3.09% of control *p* < 0.0001) was much greater than that seen with metformin (63.36% ± 6.01% of control, *p* < 0.0001) or paclitaxel (47.28% ± 3.13% of control, *p* < 0.0001) treatment (Figure 2B).

### 2.3. Effects of Rosemary Extract on Akt

**Figure 3:**
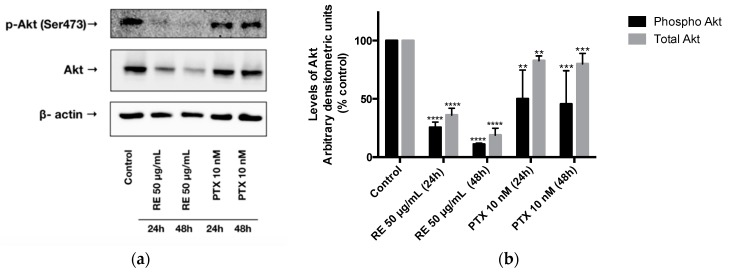
Effect of rosemary extract on Akt. Cell lysates were prepared from MDA-MB-231 breast cancer cells treated with either 50 µg/mL rosemary extract (RE) or 10 nM paclitaxel (PTX) for 24 or 48 h. Cell lysates (20 µg) were immunoblotted using specific antibodies against Akt, p-Akt, or β-actin. Representative immunoblots are shown (**a**). The densitometry of the bands, expressed in arbitrary densitometric units, was corrected to β-actin levels and is presented as percent of control (**b**). The data are the mean ± SEM of three independent experiments. ** *p* < 0.01, *** *p* < 0.001, **** *p* < 0.0001.

Next, we examined the effects of RE treatment on Akt phosphorylation/activation and expression. We measured the levels of Akt phosphorylation on the serine 473 residue, an established indicator of Akt activity [69]. Treatment of MDA-MB-231 breast cancer cells with rosemary extract (50 µg/mL) for 24 or 48 h resulted in a significant decrease in Akt phosphorylation/activation (25.62% ± 2.56% of control, *p* < 0.0001) and (11.05% ± 0.62% of control, *p* < 0.0001), respectively, (Figure 3a,b). The total Akt levels were also significantly reduced by RE treatment for 24 (36.18% ± 5.75% of control, *p* < 0.0001) and 48 h (18.89% ± 5.99% of control, *p* < 0.0001). Treatment of MDA-MB-231 cells with paclitaxel for 24 h showed a significant reduction of Akt phosphorylation/activation (50.06% ± 14.25% of control, *p* < 0.01) and total Akt levels (82.78% ± 4.02% of control, *p* < 0.01). Also, treatment of MDA-MB-231 cells with paclitaxel for 48 h resulted in a significant inhibition of Akt phosphorylation/activation (45.59% ± 16.47% of control, *p* < 0.001). The total Akt levels were also significantly reduced by paclitaxel treatment (80.047% ± 8.84% of control, *p* < 0.001) (Figure 3b). When we expressed the data as the ratio of p-Akt/total Akt we found a significant inhibition with RE treatment (RE 24 h: 71% of control (*p* < 0.05), RE 48 h: 58% of control (*p* < 0.05)).

### 2.4. Effects of Rosemary Extract on mTOR

**Figure 4:**
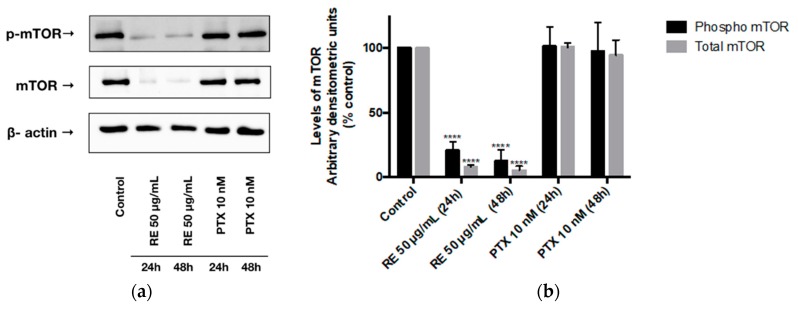
Effect of rosemary extract on mTOR. Cell lysates were prepared from MDA-MB-231 cells treated with either 50 µg/mL rosemary extract (RE) or 10 nM paclitaxel (PTX) for 24 or 48 h. Cell lysates (20 µg) were immunoblotted using specific antibodies against mTOR, phosphorylated/activated mTOR, or β-actin. Representative immunoblots are shown (**a**). The densitometry of the bands, expressed in arbitrary densitometric units, was corrected to β-actin levels and is presented as percent of control (**b**). The data are the mean ± SEM of three independent experiments. **** *p* < 0.0001.

We also examined mTOR phosphorylation/activation levels on the serine 2448 residue. Treatment of MDA-MB-231 cells with RE for 24 or 48 h resulted in a significant decrease in mTOR phosphorylation/activation (20.78% ± 3.90% of control, *p* < 0.0001) and (12.49% ± 5.04% of control, *p* < 0.0001), respectively, (Figure 4a,b). In addition, total-mTOR expression was significantly reduced by 24 h (8.11% ± 1.32% of control, *p* < 0.0001) and 48 h (5.34% ± 3.28% of control, *p* < 0.0001) RE treatment. Exposing MDA-MB-231 cells to paclitaxel for 24 h did not result in a statistically significant inhibition of mTOR phosphorylation/activation (101.35% ± 8.62% of control, *p* > 0.05) or total mTOR expression (100.55% ± 3.13% of control, *p* > 0.05). Treatment with PTX for 48 h did not result in a statistically significant reduction of either phosphorylated/activated mTOR (97.58% ± 12.53% of control, *p* > 0.05) or total mTOR (94.34% ± 11.66% of control, *p* > 0.05) levels.

### 2.5. Effects of Rosemary Extract on MDA-MB-231 Breast Cancer Cell Apoptosis

**Figure 5:**
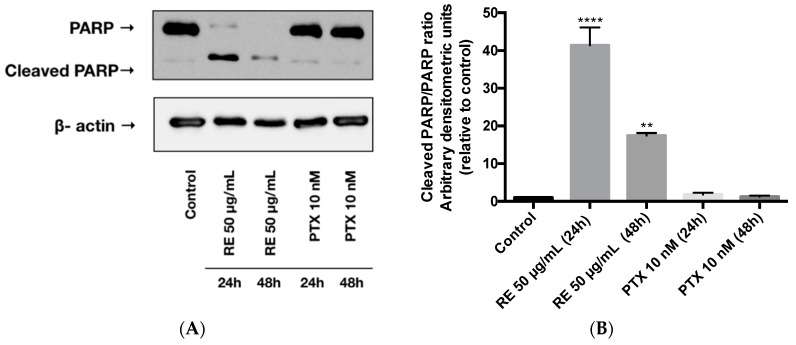
Effect of rosemary extract on PARP. Cell lysates were prepared from MDA-MB-231 cells treated with either 50 µg/mL rosemary extract (RE) or 10 nM paclitaxel (PTX) for 24 or 48 h. Cell lysates (20 µg) were immunoblotted using specific antibodies against PARP or β-actin. Representative immunoblots are shown (**A**). The densitometry of the bands, expressed in arbitrary densitometric units, was corrected to β-actin levels and is presented as a fold increase relative to control untreated cells (**B**). The data are the mean ± SEM of three independent experiments. ** *p* < 0.01, **** *p* < 0.0001.

We next examined the effect of RE on cell apoptosis by measuring the levels of cleaved PARP, an established indicator of apoptosis [39]. Exposing the MDA-MB-231 cells to rosemary extract (50 µg/mL) for 24 or 48 h resulted in a significant increase in cleaved PARP (Figure 5A,B). RE treatment for 24 or 48 h increased the cleaved-PARP/PARP ratio (41.4- and 17.5-fold increase, respectively) relative to the control untreated cells (Figure 5B). Treatment of MDA-MB-231 cells with 10 nM paclitaxel for 24 or 48 h did not result in any changes to the level of cleaved PARP (Figure 5A). The cleaved-PARP/PARP ratio in the paclitaxel-treated cells was the same as in the control untreated cells (Figure 5B).

**Figure 6:**
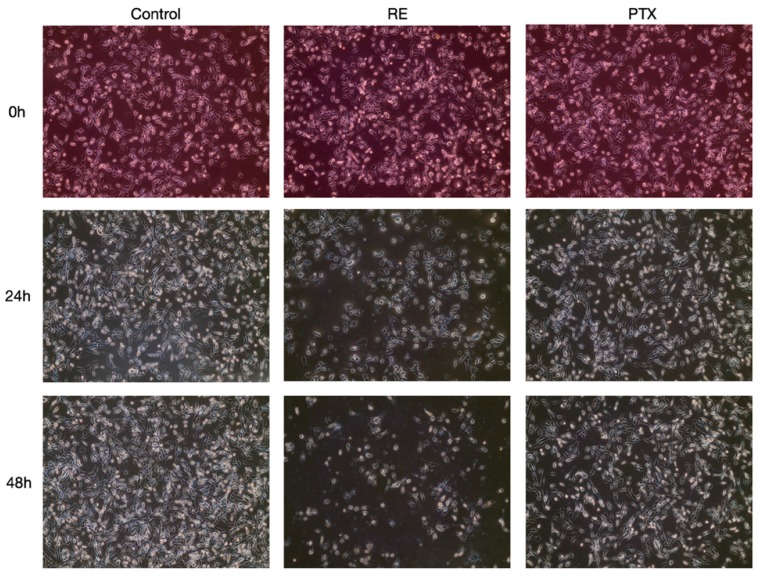
Effect of rosemary extract and paclitaxel on MDA-MB-231 cell morphology. Cells were seeded (200,000 cells/well) and after 24 h were treated without (control) or with RE (50 µg/mL) or PTX (10 nM) for 24 or 48 h. Photographs were taken immediately after treatment using an EVOS XL Core Cell Imaging System by Life Technologies (10× magnification).

We routinely examined microscopically the effect of our treatments on cell morphology. Figure 6 shows a representative image of RE- or PTX-treated cells compared to the control untreated cells. No changes in cell morphology were observed with any of the treatments. It should be noted that the same number of cells were seeded in all wells (six-well plates were used). As it can be seen from Figure 6, treatment with 50 µg/mL of RE for 24 or 48 h resulted in a substantial reduction of cell density relative to the control untreated cells. Although treatment with paclitaxel (10 nM) for 24 or 48 h reduced cell density, it did not result in a greater inhibition than RE. Furthermore, using the Bradford protein assay we measured total protein yield and found that treatment with RE significantly reduced protein yield (control, 327.3 µg/well; RE 24 h, 281.4 µg/well (*p* < 0.05 compared to control); RE 48 h, 255 µg/well (*p* < 0.05 compared to control)).

### 2.6. Effects of Rosemary Extract on MDA-MB-231 Breast Cancer Cell Migfration

**Figure 7:**
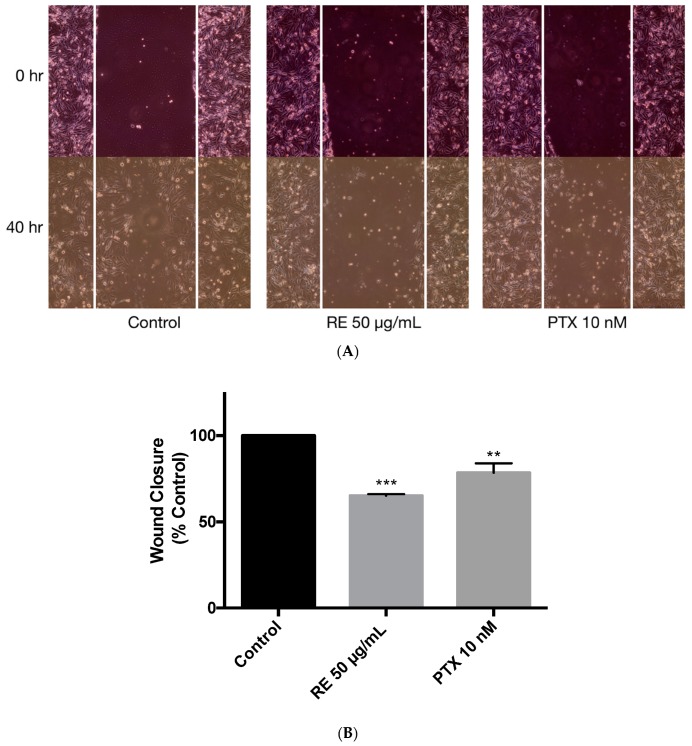
Effect of rosemary extract on MDA-MB-231 breast cancer cell migration. MDA-MB-231 cells were exposed to 1 µg/mL of mitomycin-C for one hour, followed by treatment with or without 50 µg/mL rosemary extract (RE) or 10 nM paclitaxel (PTX) for 40 h. Representative images of wound healing are shown immediately after being scratched with a 200 µL pipette tip (0 h) and after 40 h of treatment (**A**). Wound closure was calculated as indicated in the methods and expressed as a percent of control untreated cells (**B**). The data are the mean ± SEM of three independent experiments. ** *p* < 0.01, *** *p* < 0.001.

The ability of cancer cells to migrate was assessed using the wound healing assay [70,71,72]. MDA-MB-231 cells were exposed to 1 µg/mL of mitomycin-C (MMC) for 1 h to inhibit cell proliferation and were then treated with either 50 µg/mL rosemary extract or 10 nM paclitaxel for 40 h. Treatment with rosemary extract was shown to significantly inhibit wound closure (65.15% ± 0.97% of control, *p* < 0.001) suggesting that RE may negatively regulate cell migration (Figure 7B). A significant inhibition of cell migration was also seen when treating the cells with 10 nM paclitaxel (78.44% ± 5.60%, *p* < 0.01) (Figure 7A,B).

## 3. Discussion

In the present study we found a dose-dependent inhibition of MDA-MB-231 breast cancer cell proliferation with rosemary extract treatment (Figure 1). The highest inhibition of proliferation was seen at 50 µg/mL RE (34.79% ± 2.321% of control, *p* < 0.0001) with a calculated IC_50_ value of 28.86 µg/mL. Our data are in agreement with a few other studies [59,60,73,74] that have examined the effect of RE in breast cancer cells. In MCF-7 (ER positive) and MDA-MB-231 (TN) breast cancer cells, rosemary extract decreased cell viability in a dose-dependent manner, with an IC_50_ value of 20.42 µg /mL [59]. Similarly, rosemary extract dose-dependently decreased viability of five breast cancer cell lines, including MDA-MB-231 cells [60]. Apart from the above two studies [59,60] that examined the effect of RE on breast cancer cell viability, Marrelli et al. found that MCF-7 breast cancer cells treated with 100 µg/mL of RE had reduced (45% of control) proliferation [73]. A study by Telang et al. found that treating HER2 enriched 184-B5 mammary epithelial cells with 10 µg/mL of RE inhibited cell cycle progression, inhibited the G_1_ to S phase transition, and induced G_1_ phase arrest [74]. Furthermore, the RE polyphenols carnosol, carnosic acid, and ursolic acid effectively inhibited proliferation of ER positive MCF-7 breast cancer cells [74]. We have previously found an inhibition of A549 lung cancer cell proliferation by RE treatment [57] with an IC_50_ value of 15.9 µg/mL. Based on this evidence and comparing the IC_50_ values it appears that TN breast cancer cells (IC_50_ 28.86 µg/mL) are less sensitive to RE treatment than lung adenocarcinoma A549 cells (IC_50_ 15.9 µg/mL).

Apart from the suppression of cell proliferation, the present study found that treatment with RE inhibited clonogenic survival of MDA-MB-231 cells in a dose-dependent manner (Figure 2). Significant inhibition was seen at the lowest dose of 2.5 µg/mL RE (66.47% ± 7.39% of control, *p* < 0.001) and the highest inhibition was seen at 25 µg/mL RE (9.33% ± 3.09% of control, *p* < 0.0001) with a calculated IC_50_ value of 4.82 µg/mL. While no other studies have examined the effect of RE treatment on TN breast cancer cell survival, two studies using MCF-7 [60] and 184-B5/HER [74] breast cancer cells found dose-dependent inhibition of survival with RE treatment, with an IC_50_ value of 4.6 µg/mL in 184-B5/HER cells. Comparing this IC_50_ of RE (4.6 µg/mL) to the one found in the present study (4.82 µg/mL) we see almost identical values suggesting that different breast cancer cells may have a similar sensitivity to RE. It is important to note that the inhibition of MDA-MB-231 cell proliferation by RE treatment in the present study was greater than the inhibition seen with metformin or paclitaxel treatment. Metformin is derived from the plant *Galega officinalis*, commonly known as French lilac, used for many years in the management of type 2 diabetes mellitus [66] and recently studies [64,65] have provided evidence of its anticancer properties. In previous studies by our lab, we found a significant inhibition of lung cancer cell proliferation and survival by metformin treatment [63] and for this reason we used it in the current study with the intention to serve as a positive control. Metformin (5 mM) treatment did not have a significant effect on cell proliferation, while it resulted in a significant inhibition of cell survival (63.36% ± 6.01% of control) that was less than the inhibition seen with RE treatment. Metformin (4–8 mM) was previously found to reduce MDA-MB-231 breast cancer cell growth, while having no significant effect on noncancerous breast epithelial MDCK cells [75]. Similarly, metformin (0.5 and 1 mM) was shown to significantly reduce MDA-MB-231 and MCF-7 breast cancer cell viability and slightly increase cell proliferation [76]. In vivo animal studies have shown a significant reduction in tumor growth/volume in animals xenografted with 4T1 and M-406 TN breast cancer cells and treated with 2 g/L metformin added to drinking water [75]. Metformin inhibited mitochondrial respiration and reduced Ki67 expression, a marker of proliferation [75]. In addition, previous studies have shown a significant inhibition of mitochondrial complex I, activation of AMP-activated protein kinase (AMPK) [77] and inhibition of mTOR with metformin treatment of breast cancer cells [78]. Based on this evidence [77,78], together with evidence from our lab indicating activation of AMPK and inhibition of mTOR with RE in L6 muscle cells [79], we used metformin (5 mM) in our study with the intention to act as a positive control and compare the mechanism of action of RE to that of metformin. In contrast to the above studies, we did not find any significant effect on cell proliferation with metformin treatment. Similar to our findings and in contrast to the above-mentioned findings [75,76] treatment of MDA-MB-231 breast cancer cells with metformin (8 mM) did not affect cell proliferation or apoptosis [80,81]. Overall, the effect of metformin on breast cancer cells and MDA-MB-231 cells specifically are not consistent across different studies. The reasons for these discrepancies are not clear and we recognize that the use of other agents, such as taxanes or doxorubicin, may have been more appropriate positive control than metformin.

We also wished to compare the effect of RE treatment with that of an agent used clinically to treat TN breast cancer and we used paclitaxel. Specific chemotherapeutic agents targeting TN breast cancer do not exist and this type of cancer is often treated using paclitaxel [68,82,83]. Cell viability and proliferation of TN breast cancer cells was decreased with paclitaxel treatment in previous studies [60,83]. TN breast cancer patients treated with paclitaxel have serum levels of paclitaxel in the nanomolar (12 nM) [68] to the micromolar (3.3 µM) [67] range. In the present study we found a significant inhibition of proliferation and survival with 2–10 nM paclitaxel treatment. We used these concentrations of paclitaxel based on previous in vitro studies [60,83] and taking into account the paclitaxel serum levels in treated patients [67,68]. Importantly, the effects of RE treatment were greater than the effect of paclitaxel, and these data suggest a strong anticancer potential of RE that justifies further studies.

The effect of RE on signaling pathways that control MDA-MB-231 cell proliferation and survival has not been studied previously. Akt controls cellular growth and survival/apoptosis [84] and its activation is increased in cancer cells, including breast cancer [11,15,84,85]. It should be noted that the levels of phosphorylated Akt (Figure 3) in the control untreated MDA-MB-231 cells are high indicating that its overactivation can possibly drive proliferation and survival. Our observation is in agreement with other studies that have shown that the levels of phosphorylated/activated Akt [86] are high in TN breast cancer. Importantly, our study is the first to show that treatment with 50 µg/mL RE significantly inhibited phosphorylation/activation of Akt (Figure 3) in MDA-MB-231 cells. The recognition of enhanced Akt signaling in cancer, including breast cancer [87], has led to the development of novel agents targeting Akt, such as perifosine, BAY1125976, MK-2206, afuresertib, miransertib, and ipatasertib, all currently in clinical trials [88]. Some of these Akt-targeting agents are classified as allosteric inhibitors (MK-2206) while others (ipatasertib) are ATP-competitive inhibitors [89]. RE has a potent inhibitory effect on Akt, but the mechanism involved is not known. It is possible that RE contains chemicals that act as APT-competitive or allosteric Akt inhibitors. Alternatively, chemicals present in RE may influence steps upstream of Akt such as inhibition of PI3K or increased expression and activation of PTEN. The inhibition of Akt by RE deserves attention and further investigation.

In addition to Akt, mTOR, a signaling molecule that promotes protein synthesis, cell proliferation and survival, is often activated in cancer due to mutations that are found upstream of mTOR itself, such as a gain-of-function mutation of PI3K and loss-of-function mutations on the tumor suppressor gene PTEN [26]. The activation of mTOR increases the rate of protein synthesis and suppresses autophagy in cancer cells [38]. It has been shown that activated mTOR leads to increased tumor progression and decreased patient survival, with mTOR activation being more common in TN breast cancer [33,38]. In agreement with this evidence, we observed that the phosphorylation/activation levels of mTOR in the control untreated MDA-MB-231 cells (Figure 4) were high, indicating its overactivation. The evidence of enhanced mTOR signaling in cancer led to the development of novel mTOR inhibitors that are used in cancer treatment [16,24,25,26,27,32]. Our data show a very potent inhibition of mTOR with RE treatment. It is possible that this inhibition of mTOR is due to the inhibition of Akt, an upstream activator of mTOR, or inhibition of any other signaling steps involved in mTOR activation.

We observed potent inhibition of both Akt and mTOR phosphorylation/activation with RE treatment in the present study. As mentioned above, effective inhibitors of Akt, mTOR, or both are highly desired and employed in the treatment of TN breast cancer [34,90]. An early-phase clinical trial showed that ipatasertib, an Akt inhibitor, combined with paclitaxel improved the progression-free survival of patients with TN breast cancer compared to paclitaxel alone (6.2 vs. 4.9 months, *p* = 0.037) [91]. Another phase II study showed that the combination of ipatasertib (400 mg daily) and paclitaxel (80 mg/m^2^ weekly) was well tolerated by women with TN breast cancer [21]. The mTOR inhibitor everolimus was shown to increase the effectiveness of paclitaxel in treating TN breast cancers in clinical trials [34]. The inhibition of total Akt and mTOR levels with RE treatment (Figure 3 and Figure 4) suggests that this reduction could be due to the inhibition of gene transcription, inhibition of protein synthesis, or increased protein degradation. RE may possibly have an effect on protein stability. The inhibition of total Akt protein and mRNA levels has previously been reported in K562 leukemia cells treated with 50 µg/mL RE for 24–48 h [92]. The same study also found that terpinolene, a constituent of rosemary and sage, among some of the main constituents of rosemary tested, reduced Akt protein expression in K526 cells [92]. Additionally, a previous study by our group showed a significant reduction of total Akt and mTOR expression by 50 µg/mL RE treatment in A549 lung cancer cells [57]. It is important to note that the inhibitory effects of rosemary extract on Akt and mTOR observed in the present study are similar to the effects of perifosine, a novel inhibitor of Akt, currently in clinical trials. Perifosine inhibited Akt phosphorylation and reduced total Akt levels in H157, H460, and A549 lung cancer cells [93]. Similarly, in HCT116 human colon cancer cells, perifosine inhibited Akt phosphorylation and reduced total Akt, mTOR, and p70S6K levels, resulting in the induction of apoptosis and autophagy [94]. Previously it was shown that treatment of MDA-MB-231 cells with the polyphenol curcumin resulted in significant inhibition of cell proliferation and migration that correlated with Akt degradation [95]. Treatment with curcumin induced autophagy, activated AMPK, and suppressed the ubiquitin-proteasome system [95]. This study indicates that the activation of AMPK could lead to activation of the autophagy–lysosomal protein degradation pathway resulting in Akt degradation [95]. It is possible that the reduced total Akt levels seen with RE treatment in our study may be due to activation of a similar pathway and requires further study. 

We searched the scientific literature to find other stimuli/treatments that inhibit total Akt levels in breast cancer and we found salinomycin, a drug used originally to eliminate bacteria and parasites. Similar to our data, treatment of Hs578T breast cancer cells with salinomycin alone or in combination with MK-2206, an allosteric Akt inhibitor, resulted in reduced phosphorylated and total Akt levels [96].

Total mTOR protein levels are elevated in breast cancer compared to normal cells [97]. Although mTOR inhibitors have shown promise as anticancer agents, there is a high risk of drug resistance [97]. Inhibition of mTOR initiates a feedback loop that upregulates upstream receptor tyrosine kinases, which activate Akt and reactivate mTOR, limiting the effectiveness of these inhibitors [36,98]. This feedback regulation of mTOR involves increased levels of total mTOR and is a suggested mechanism of drug resistance [97]. Thus, utilizing an agent that will inhibit the expression of the mTOR protein, in addition to the inhibition of mTOR activation, may potentially improve the efficacy of breast cancer treatments and decrease drug resistance [97]. Similar to our data, it was shown that MCF-7 breast cancer cells treated with metformin and rapamycin, two known mTOR inhibitors, had decreased levels of both total-mTOR and phosphorylated/activated mTOR [97]. Overall, utilizing agents that target both Akt and mTOR, causing both a reduction in the phosphorylated/activated and total levels of these proteins, may result in cancer cells becoming more sensitized to treatments and reducing drug resistance. The targeted degradation of Akt and mTOR in cancer may open new avenues and improve efficacy of treatment.

RE also enhanced the level of cleaved PARP (Figure 5), a well-established marker of apoptosis [39]. In contrast, treatment of the cells with paclitaxel did not induce PARP cleavage, suggesting that different mechanisms are involved in the anticancer effects of RE and paclitaxel. Similar to our findings, treatment of MDA-MB-231 cells with 10 nM paclitaxel did not induce PARP cleavage [99]. Interestingly, PARP cleavage was seen with 20 and 200 nM paclitaxel treatment (48 h) of MDA-MB-231 cells in a recent study by Shetti et al. [100]. The differences between our data, the data by Calaf et al. [99] and the data by Shetti et al. [100] may be due to the concentration of PTX used (10 nM vs. 20–200 nM ). Our data are similar to the study by Calaf et al. where PARP cleavage was seen with the polyphenol curcumin but not with PTX treatment of MDA-MB-231 cells [99].

A previous study by our group also found a two-fold increase in PARP cleavage in A549 non-small-cell lung cancer cells treated with RE [57]. Similar to our findings, treatment of MDA-MB-231 cells with carnosol (COH), a polyphenol found in RE, resulted in increased apoptosis, as indicated by the increased PARP cleavage and increased cleaved caspase 3, 8, and 9 levels [101]. COH was also seen to increase the expression of the proapoptotic protein Bax and decrease the antiapoptotic protein Bcl2 [101]. It is possible that the proapoptotic effect seen with the RE treatment in our study may be due to COH or other polyphenols found in RE. Previously, using high-performance liquid chromatography (HPLC), we measured the levels of the polyphenols carnosic acid (CA) and rosmarinic acid (RA) in our extract and found them to be 2.12% ± 0.22% [102] and 13.39% ± 0.23% [103], respectively. Taking into account the molecular weight of CA (332.42 g/mol) and RA (360.31 g/mol) we calculated that in media containing 50 μg/mL of RE, the concentration that resulted in maximum inhibition of cell proliferation, the corresponding concentrations of CA and RA are 3 μmol/L and 20 μmol/L, respectively. Our aim is to identify in the future the component(s) of RE that have strong anticancer effects. We have already initiated our studies by investigating the effects of CA, RA, and COH (no data available yet). However, a few in vitro studies have shown that CA, RA, and COH inhibited cell proliferation and induced apoptosis in various breast cancer cell lines [59,101,104,105]. Specifically, CA was shown to inhibit proliferation in ER negative human breast cancer cells by inducing G1 cell cycle arrest [104]. Treatment with CA (19 µM) showed 70% inhibition of MCF-7 breast cancer cell viability, while a two-fold greater inhibition was seen in the estrogen independent TN MDA-MB-231 breast cancer cells [59]. Similarly, COH reduced the cell viability of MDA-MB-231 breast cancer cells in a dose-dependent manner with an IC_50_ value of 83 µM [101]. Interestingly, RA did not inhibit proliferation of MDA-MB-231 breast cancer cells [59] suggesting that this polyphenol may not be responsible for the antiproliferative effects shown by RE treatment. Based on these studies, we hypothesize that the polyphenols CA and COH may be responsible for the observed effects of RE in the present study.

Our study showed that treatment with RE significantly inhibited MDA-MB-231 cell migration (Figure 7). Although no other studies exist examining the antimigratory or antimetastatic effects of RE in TN breast cancer cells, one study found a significant inhibition of MDA-MB-231 cell migration when treated with the rosemary polyphenol CA [105]. In addition, the inhibition of cell migration seen with CA in combination with trastuzumab, a chemotherapeutic agent used in clinical practice, resulted in a greater inhibition of cell migration when compared to each agent alone as a monotherapy [105]. These data indicate a chemo-sensitive effect of RE polyphenols.

In our study we have observed a significant inhibition of cell proliferation, cell migration, and inhibition of total and phosphorylated Akt levels. There are three Akt isoforms (Akt1, Akt2, Ak3) found in mammalian cells, each with a distinct role in cancer. Akt1 may promote cell proliferation, while Akt2 is responsible for regulating cell migration and invasion [106,107]. The contribution of each individual Akt isoform in the inhibition of cell proliferation and migration seen in our study is not clear. More studies should be performed in the future to elucidate the specific role of each individual Akt isoform.

There are no studies examining the effects of RE treatment using TN breast cancer xenograft models. In one study, intraperitoneal injections of RE or COH at 200 mg/kg for 5 days in female rats inhibited the DMBA-induced mammary adduct formation (by 44% and 40%, respectively) [108]. In another study, administration of CA to mice inoculated with ER positive breast cancer cells resulted in significant inhibition of tumor growth [109]. Furthermore, treatment of mice xenografted with ER positive breast cancer cells with CA and tamoxifen (30 and 10 mg/kg, respectively) as a combined therapy resulted in a greater inhibition of tumor growth in comparison to CA or tamoxifen monotherapy [109]. Although the studies examining the effects of rosemary extract and rosemary extract polyphenols in vivo are limited, the available evidence suggest that they may be effective in inhibiting tumor growth as a monotherapy or as a combined therapy with other chemotherapeutic agents. In vivo animal studies utilizing TN breast cancer xenograft models are required to better understand the effects of RE and RE polyphenols against this subtype of cancer.

## 4. Materials and Methods

### 4.1. Materials

The MDA-MB-231 human epithelial breast cancer cells were obtained from American Type Culture Collection (ATCC) (Manassas, VA, USA). The Dulbecco’s Modified Eagle’s Medium (DMEM), fetal bovine serum (FBS), 0.25% trypsin and the antibiotic–antimycotic solution were purchased from GIBCO Life Technologies (Burlington, ON, Canada). Akt (#9272) (1:1000 dilution), p-Akt (Ser473) (#9271) (1:1000 dilution), mTOR (#2972) (1:1000 dilution), p-mTOR (Ser2448) (#2971) (1:1000 dilution), PARP (#9542) (1:1000 dilution), β-actin (#8457) (1:1000 dilution), as well as secondary anti-rabbit IgG HRP-linked antibodies (#7074) (1:2000 dilution) were from Cell Signaling Technology via New England Biolabs (Mississauga, ON, Canada). Bovine serum albumin (BSA), dimethyl sulfoxide (DMSO), Paclitaxel, and Metformin were from Sigma (Oakville, ON, Canada). Clarity Western enhanced chemiluminescence substrate (ECL), 30% acrylamide/bis solution 37 (5:1), ammonium persulfate (APS), polyvinylidene difluoride (PVDF) membranes and reagents for electrophoresis were purchased from Bio-Rad (Hercules, CA, USA).

### 4.2. Rosemary Extract Preparation

Whole dried rosemary (*Rosmarinus officinalis* L.) leaves (purchased from Compliments/Sobey’s, Mississauga, ON, Canada) were used, and the rosemary extract was prepared as previously reported [57]. Briefly, dried rosemary leaves were ground and steeped overnight (16 h) in dichloromethane:methanol (1:1) followed by filtration the next day. After filtering, the solvent was set aside while the leaves were boiled in methanol for 30 min. The solvent obtained after boiling was combined with the filtered solvent. The combined solvent was removed from the final extract by rotary evaporation and the green powder was collected and stored at −20 °C, protected from light. Aliquots were prepared in dimethyl sulfoxide (DMSO) to yield a final concentration of 100 mg/mL.

### 4.3. Cell Culture and Treatment

The cells were cultured in DMEM media supplemented with 1% (*v*/*v*) antibiotic–antimycotic solution (containing 100 units/mL of penicillin, 100 µg/mL of streptomycin, and 0.25 µg/mL of Amphotericin B) and 10% (*v*/*v*) FBS in an incubator at 37 °C.

Cells were treated with a working stock of RE (400 µg/mL in cell culture media) and the final concentration of DMSO in the RE-treated cells was less than 0.1%. Exposure of the cells to DMSO to match the concentration of DMSO seen by cells exposed to RE (vehicle control) did not have any effect on any assays/measurements used in the current study.

### 4.4. Cell Proliferation Assay

Cells were seeded (1000 cell/well) in a 96-well plate supplemented with DMEM and treated as indicated in the figures for 72 h. The cells were fixed with 10% formalin and stained using 0.5% crystal violet stain. The next day solubilizer solution containing 0.05 M NaH_2_PO_4_ was added into each well and the absorbance was read at 570 nm using the KC4 microplate reader.

### 4.5. Clonogenic Survival Assay

Cells were seeded (1000 cells/well) in six-well plates and allowed to adhere for 24 h followed by treatment as indicated in the figures for seven days. At the end of the treatment, the cells were washed twice with sterile phosphate-buffered saline (PBS) and stained with 0.05% *w*/*v* methylene blue. The next day, colonies greater than 50 cells were counted under the microscope.

### 4.6. Immunoblotting

Cell lysate samples containing 20 µg of protein, determined using the Bradford assay [110], were loaded onto 10% polyacrylamide gel and separated by sodium dodecyl sulfate–polyacrylamide gel electrophoresis (SDS-PAGE). The separated proteins were then transferred onto polyvinylidene difluoride (PVDF), membrane which was exposed to blocking buffer (5% (*w*/*v*) dry milk in Tris-buffered saline) for 1 h and incubated with the primary antibody overnight at 4 °C. The following day the membrane was incubated with horseradish peroxidase (HRP)-linked IgG anti-rabbit secondary antibody for 1 h at room temperature. Enhanced chemiluminescence (ECL), the Bio-Rad Clarity Western solution, was used to detect the bands corresponding to the proteins of interest. Densitometric analysis was performed using ImageJ software. The data (arbitrary densitometric units) were corrected to β-actin levels and expressed as a percentage of untreated control cells.

### 4.7. Statistical Analysis

The data are the mean ± standard error mean (SEM) of the indicated number of independent experiments. Analysis of variance (ANOVA) followed by Bonferroni’s post-hoc test was used to determine the significance of the differences between groups. Significance was assumed at *p* < 0.05. Statistical tests were performed using GraphPad Prism software.

## 5. Conclusions

**Figure 8:**
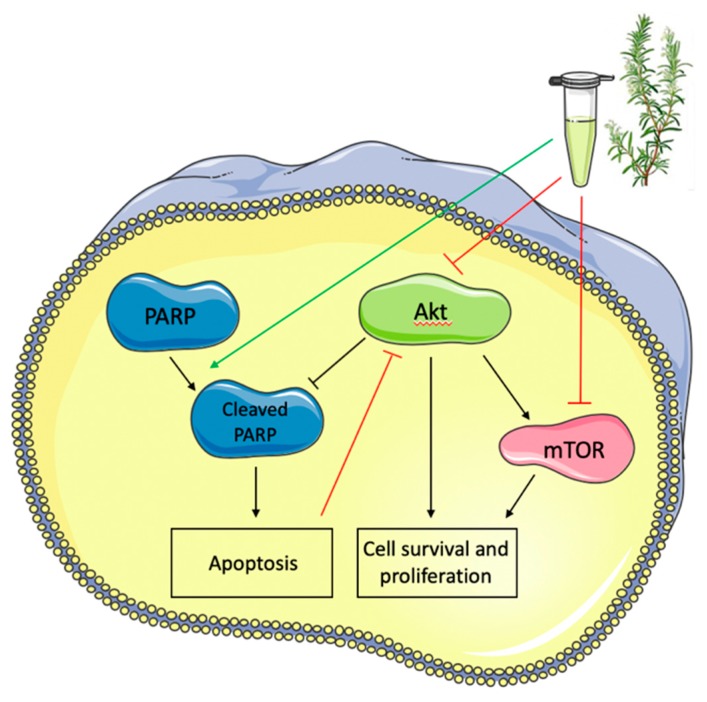
Effects of rosemary extract on MDA-MB-231 triple-negative breast cancer cell signaling molecules. RE inhibited the phosphorylation/activation and total levels of Akt and mTOR, while it enhanced the levels of cleaved PARP.

Breast cancer is the most commonly diagnosed cancer and the second leading cause of cancer-related death among women worldwide. Triple-negative breast cancer is one of the most aggressive subtypes of breast cancer, and does not respond to conventional hormone therapy, therefore innovative treatments are being sought. The present study is one of the few studies that have examined the effects of rosemary extract on MDA-MB-231 triple-negative breast cancer cells. We found that RE significantly inhibited MDA-MB-231 cell proliferation, and survival (Figure 8). The levels of cleaved PARP, an established marker of cellular apoptosis, were increased by RE treatment (Figure 8). In addition, RE significantly reduced the phosphorylation/activation and total levels of Akt and mTOR, key players controlling cancer cell proliferation and survival (Figure 8).

In the present study only one TN breast cancer cell line, the MDA-MB-231, was used. These cells are characterized by a p53 and Ras mutation [111]. We recognize that this is a limitation and strongly recommend that more studies should be performed in the future utilizing other cell lines representing TN breast cancer to better understand and elucidate the signaling mechanisms involved.

Although the present study indicates that the effects of RE were comparable to the effects of paclitaxel, a chemotherapeutic agent used in the treatment of TN breast cancer, more studies examining the effect of RE in combination with classical chemotherapy agents against TN breast cancer such as taxanes or anthracyclines should be performed.

Our data indicate that RE has potent anticancer effects in MDA-MB-231 cells and may have potential to be used in cancer treatment. Further research should investigate (a) the exact polyphenolic constituent(s) of rosemary extract that contribute to its anticancer effects and (b) the mechanism of action of RE and RE polyphenols on other breast cancer cells and xenograft animal models.
